# Observations on Lithotomy, Lithotrity and the Early Detection of Stone in the Bladder, with a Description of a New Method of Tapping the Bladder

**Published:** 1884-03

**Authors:** 


*Observations on Lithotomy, Lithotrity and the early detection of Stone in the Bladder, with a description of a new method of tapping the Bladder.* By REGINALD HARRISON, F.R.C.S. Pp. 71. London: J. & A. CHURCHILL, 1883.

The honestly recorded experience of a practical, pains-taking and independent surgeon is always welcome. And the record is doubly welcome when, as in this case, it is made in a simple, straightforward and concise manner, dwelling only on those points which the author considers noteworthy or has made specially his own, and passing over others which are well established and generally understood.

In the operation of lithotomy the author tells us that he adheres as closely as possible to the lines laid down by Cheselden, and gives additional short directions as to how to meet the chief difficulties which may arise. He introduces a tube into the bladder after the operation, as it prevents obstruction by clots to the free flow of the urine, and keeps the patient drier and more comfortable. When the perineum is deep, or the prostate large, he has found the cutting gorget of service.

The most important portion of the work relates to lithotrity and the revolution recently introduced in its methods by Professor Bigelow. We infer that Mr. Harrison considers that rapid lithotrity has removed from the domain of lithotomy stones of almost any size, not only in the adult and in moderately old people, but also in children of not too tender an age, where the stones are under half-an-inch in the longest diameter. It has been said that we do not want a better operation than lithotomy for children; but if a stone three-eights of an inch in diameter can be removed by lithotrity from a child, and the patient sent home well in a week, we must conclude that a better operation has been found. May lithotrity and rapid evacuation of the fragments by aspiration not be expected year by year to be applied to children of increasingly tender years? If so, and if stones are detected earlier, we fear that the grand old operation of lithotomy will ere long be a rare one in surgery.

The work concludes with a description of a method of tapping the bladder through an enlarged prostrate by means of a new trocar and canula, to which a rubber tube is attached. The method has had good results in his hands and promises to be useful.

